# Case report: Abscopal response and reversal of PD-1 resistance in a patient with nephroblastoma following radiofrequency ablation

**DOI:** 10.3389/fonc.2025.1640409

**Published:** 2025-10-08

**Authors:** Mengyang Ju, Mingjuan Sun, Wenfeng Li, Sheng Zhang

**Affiliations:** ^1^ Department of Radiation Oncology, Shanghai Cancer Center, Fudan University, Shanghai, China; ^2^ Department of Biochemistry and Molecular Biology, College of Basic Medical Sciences, Naval Medical University, Shanghai, China; ^3^ Department of Medical Oncology, The Affiliated Hospital of Qingdao University, Qingdao, China; ^4^ Medical Oncology, Shanghai Cancer Center, Fudan University, Shanghai, China

**Keywords:** apatinib, camrelizumab, radiofrequency ablation, nephroblastoma, PD-1 resistance, reversal

## Abstract

Nephroblastoma (Wilms tumor, WT) is an extremely rare and aggressive malignancy in adults with nonspecific clinical and imaging features. There is no standard therapy for patients with progressive disease despite surgery and chemotherapy. Here, we report a unique case of a 27-year-old male patient with recurrent metastatic nephroblastoma who developed resistance to PD-1 inhibitor and targeted therapy. Radiofrequency ablation (RFA) was performed on the largest porta pulmonic lesion. Notably, 3 months post-ablation, a non-ablated pleural lesion exhibited a partial response. Follow-up confirmed PR of the pleural lesion and total disappearance of pleura-irritative symptoms. This case demonstrates a potential abscopal effect induced by RFA, in which local treatment of one tumor site coincided with systemic regression of distant, untreated lesions and reversal of prior PD-1 inhibitor resistance.

## 1 Introduction

Nephroblastoma (Wilms tumor, WT) is commonly diagnosed in children (1). It is an extremely rare tumor in adults, with an incidence of fewer than 0.2 per million per year (2). WT usually presents with abdominal or flank pain and is clinically indistinguishable from more common adult malignant renal neoplasms such as renal cell carcinoma (3). As a result, most cases are identified at advanced stages, frequently with local invasion or metastatic spread. Due to the rarity of this tumor in adults, there are no firmly established treatment regimens (4). Although histological differences between children and adults are insignificant, the prognosis of adult WT compared with pediatric WT is much poorer (5). Current treatment for adult patients largely follows children’s WT treatment protocols with some changes (2, 5). Recently, immune checkpoint inhibitor (ICI) therapy and targeted therapy have shown antitumor activity in various cancers, including renal cell carcinoma (6, 7), but little is known about their effect in WT. Here, we report an adult patient with WT who relapsed after achieving partial response (PR) to combined immunotherapy and targeted therapy. Radiofrequency ablation (RFA) of a single progressive lesion not only overcame resistance to immunotherapy but also induced abscopal regression of an additional metastatic lesion.

## 2 Case description

**Figure 1:**
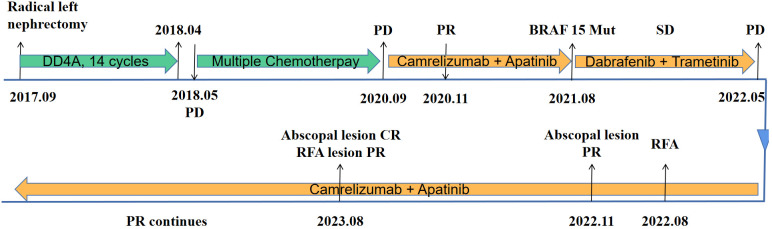
Timeline of the patient’s disease course. DD4A, vincristine, actinomycin-D, and doxorubicin (VAD) chemotherapy according to the COG DD4A protocol. CR, complete response. PR, partial response. SD, stable disease. PD, progressive disease. RFA, radiofrequency ablation.

A 27-year-old male patient was diagnosed with a left renal mass 7 years earlier. His treatment history is shown in **Figure 1**. After a confirmed diagnosis of nephroblastoma, he underwent a left radical nephrectomy. Postoperatively, the patient received 14 cycles of DD4A chemotherapy (vincristine, actinomycin-D, and doxorubicin [VAD]) according to the Children’s Oncology Group (COG) DD4A protocol. One month after the completion of chemotherapy, routine imaging surveillance revealed metastatic lesions in both lungs. Over the following 2 years, the patient was treated with multiple chemotherapy regimens, but the pulmonary lesions eventually progressed, leading to a persistent cough. In September 2020, the patient started treatment with camrelizumab (a PD-1 inhibitor) in combination with apatinib, achieving a partial response (PR) that lasted for 9 months.

**Figure 2:**
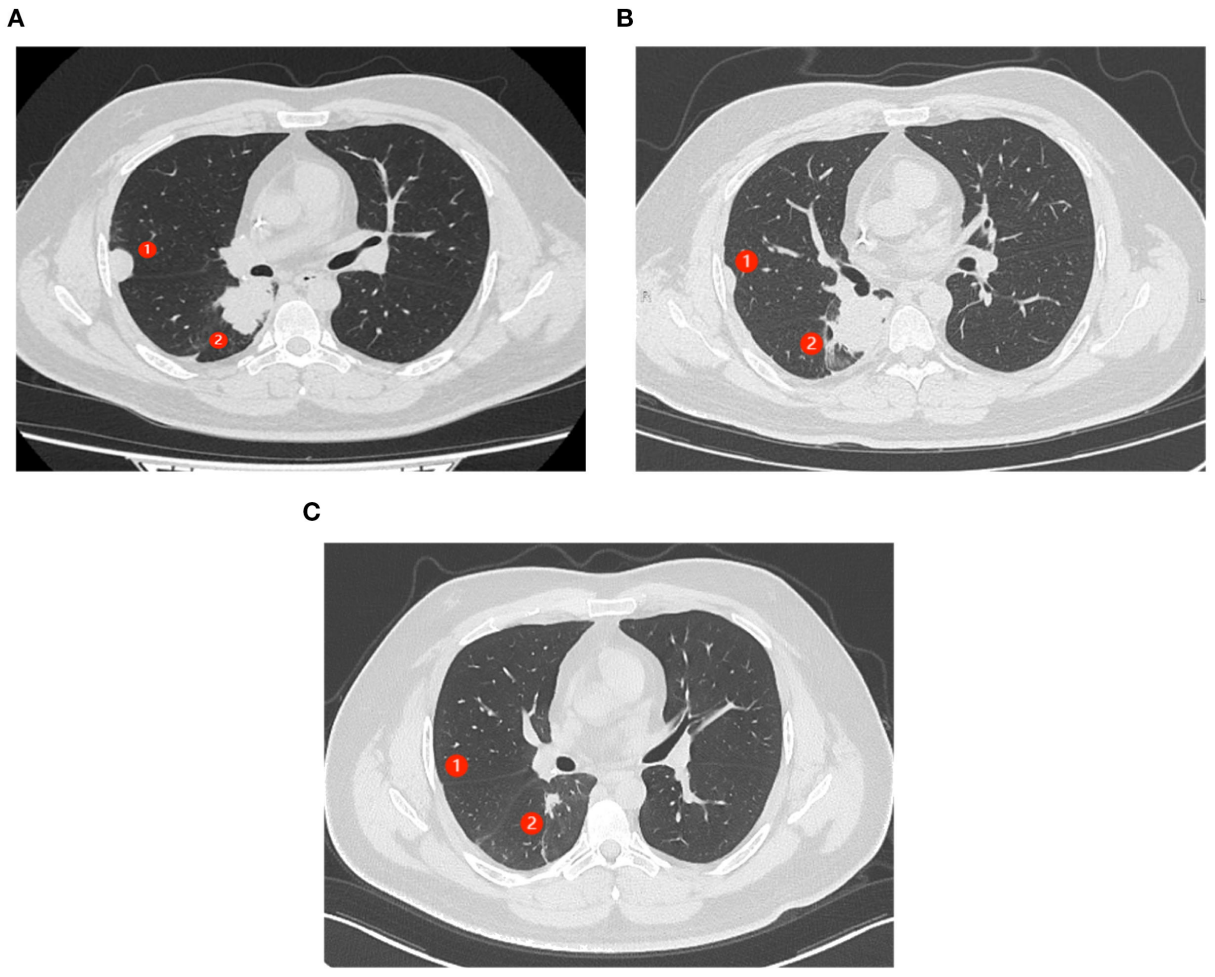
Abscopal response in a patient with nephroblastoma following radiofrequency ablation (RFA). ① represents the lesion near the chest wall that did not undergo RFA. ② represents the lesion in the hilar region where RFA had been performed. **(A)** CT scans of the patient’s chest show progressing lung lesions before the initiation of RFA. **(B)** CT scan shows the resolution of ① after 3 months of therapy (C). **(C)** CT scans (axial views) show complete response (CR) of ① and partial response (PR) of ② after 12 months of therapy.

After resistance to the PD-1 inhibitor developed, genetic testing of peripheral blood and tumor tissue identified a vrafmurine sarcoma viral oncegene homolog B (BRAF) mutation in exon 15. Based on this finding, in August 2021, the patient commenced treatment with dabrafenib and trametinib. Computed tomography (CT) assessment indicated stable disease (SD), and his cough improved. Nine months later, he reported worsening cough and persistent right-sided chest pain. CT revealed a 2-cm pleural lesion (**Figure 2A**). The patient stopped BRAF-targeted therapy and was rechallenged with camrelizumab (200 mg, every 21 days) and apatinib (250 mg, once daily). Three months later, the pulmonary lesions remained stable. He then underwent RFA of the largest lesion in the hilar region of the right lung. The longest diameter of the target lesion was approximately 4.39 cm. Only partial ablation was achieved, as the current criterion for complete RFA applies to lesions 3 cm or smaller. Three months post-RFA, CT evaluation confirmed a PR in another pleural lesion that had not undergone RFA (**Figure 2B**). The patient continued combined treatment, and at 12 months post-RFA, CT evaluation demonstrated complete response of the pleural lesion and regression of the hilar lesion that had undergone RFA (**Figure 2C**). His chest pain resolved, and his cough diminished. Serial follow-up documented a sustained partial response. Camrelizumab was discontinued in July 2025, and the patient is currently maintained on oral apatinib therapy (250 mg once daily). Treatment-related toxicities have remained generally tolerable (**Table 1**).

**Table 1:** Adverse events.

TARE	Grade
Pleural pain	2
Vomit	2
Hyperglycemia	3
Diarrhea	2
Lower-limb purpura	1
Anorexia	1
Oral ulceration	2

TARE, treatment-related side effects.

## 3 Discussion

WT is a rare malignant renal neoplasm in adults (8). Diagnosis of nephroblastoma in adults is challenging because its clinical presentation overlaps with other renal masses and no pathognomonic imaging findings exist (9). The most frequent manifestations are flank pain, gross hematuria, and abdominal mass. Cross-sectional imaging (CT or MRI) readily detects a renal mass but cannot reliably distinguish it from renal cell carcinoma; thus, definitive diagnosis rests on postsurgical histopathology. Compared with childhood Wilms tumor, the adult counterpart carries a markedly worse prognosis, presumably due to advanced stage at detection, lower chemosensitivity, and frequent comorbidities (5). Because of the rarity of adult cases, therapeutic strategies remain investigational and warrant prospective study.

Evidence suggests that PD-1/PD-L1 expression in nephroblastoma is associated with poor prognosis, but the role of PD-1 inhibitors in its treatment remains unclear (10). Previous studies have indicated that one cause of resistance to PD-1/PD-L1 therapy is the lack of highly immunogenic tumor-specific antigens, which prevents tumor cells from being recognized by T cells (11). Furthermore, tumor cells can induce the surrounding microenvironment to suppress antitumor immunity, with immunosuppressive cells, cytokines, and tumor metabolites acting as extrinsic factors that contribute to resistance (12). By triggering the release of tumor-specific antigens and reshaping the tumor microenvironment, RFA is emerging as a promising approach to overcome immunotherapy resistance (13).

In the present case, RFA was performed primarily to relieve symptoms. Several studies have demonstrated that RFA is a feasible and safe treatment for various cancers, such as thyroid nodules (14), hepatocellular carcinoma (15), and hilar cholangiocarcinoma (16). RFA is a minimally invasive modality that induces local coagulative necrosis while concurrently releasing danger-associated molecular patterns (DAMPs) capable of activating both innate and adaptive immunity (17). Nevertheless, the immune response elicited by RFA alone is typically subtherapeutic. Consequently, RFA combined with immunotherapy has emerged as a strategy to eradicate residual tumor cells and prevent post-ablative recurrence (18).

Interestingly, the lesion on the right chest wall, which did not undergo RFA, regressed and achieved a complete response to camrelizumab and apatinib. This may represent a possible abscopal effect of RFA, given the patient’s prior progression on these drugs. The role of RFA in releasing tumor-specific antigens and activating systemic immunity has garnered increasing attention (19): RFA can cause elevation in proinflammatory cytokines, which appear within hours after ablation and persist for several days. It can also reduce the number of Treg (CD4+CD25+Foxp3+) cells, diminish inhibitory immune responses, and promote antitumor immunity (20). Furthermore, studies have demonstrated that thermal ablation paired with ICI can synergistically amplify antitumor immunity (21, 22), and RFA is expected to produce comparable benefits.

In conclusion, this case highlights the potential of RFA to induce abscopal effects and reverse resistance to PD-1 inhibitors. Further clinical trials are warranted to validate this strategy.

## Data Availability

The raw data supporting the conclusions of this article will be made available by the authors, without undue reservation.
